# Can the Generalist Predator *Calosoma chinense* Kirby Be Effectively Employed in the Biological Control of *Spodoptera frugiperda* (J. E. Smith)?

**DOI:** 10.3390/insects16050437

**Published:** 2025-04-22

**Authors:** Caihong Tian, Jianrong Huang, Junyi Zhang, Guoping Li, Xuezheng Hao, Lin Wang, Xinming Yin, Hongqiang Feng

**Affiliations:** 1 Institute of Plant Protection, Henan Key Laboratory of Crop Pest Control, Key Laboratory of Integrated Pest Management on Crops in Southern Region of North China, Ministry of Agriculture and Rural Affairs of the People’s Republic of China, International Joint Research Laboratory for Crop Protection of Henan, Biological Pesticides Engineering Research Center of Henan Province, Henan Academy of Agricultural Sciences, Zhengzhou 450002, China; caihongtian@126.com (C.T.);; 2 College of Plant Protection, Henan Agricultural University, Zhengzhou 450002, China; 3 Henan Province Economic Crop Promotion Station, Zhengzhou 450002, China; jjzw2023@163.com; 4 Pherobio Technology Co., Ltd., Beijing 101102, China; wl8834@126.com

**Keywords:** *Calosoma chinense* Kirby, *Spodoptera frugiperda* (J. E. Smith), predation capacity, predation choice

## Abstract

*Calosoma chinense* Kirby, a generalist predator, consumes a variety of pests. To better understand its predatory behavior and capacity, particularly against *Spodoptera frugiperda* (J. E. Smith), we identified the field species and investigated the predation capacity of the third-instar larvae and adults of both sexes against the first- to sixth-instar larvae, pupae, and male and female adults of *S. frugiperda*. The predation preference for different developmental stages of *S. frugiperda* was further examined. Our results indicated that *C. chinense* can prey on *S. frugiperda* in various stages, including larvae and adults, demonstrating robust predatory abilities. Among them, female adults of *C. chinense* exhibited the strongest predatory ability against first-instar larvae of *S. frugiperda*, with the highest daily consumption of 38.90 ± 0.79 individuals. The first-instar larvae of *C. chinense* also showed a certain predatory ability towards *S. frugiperda*, with a daily consumption of 6.95 ± 0.88 individuals per predator. The predation ability of *C. chinense* larvae increased with larval growth, especially for the first to third instar of *S. frugiperda* larvae, and *C. chinense* female adults had the strongest predation ability, followed by male adults and then third-instar larvae. However, there was no significant difference in predation capacity against fourth- to sixth-instar larvae and male and female adults of *S. frugiperda,* nor between adults of both sexes and third-instar larvae of *C. chinense*. The predation choice experiment revealed that the third-instar larvae and male and female adults of *C. chinense* showed a positive preference for *S. frugiperda* larvae from the first to fourth instars, while for the fifth- to sixth-instar larvae, they exhibited a negative preference. Overall, *C. chinense* exhibited a strong ability to prey on *S. frugiperda.* Our study indicates that *C. chinense* has the potential for application in the biological control of *S. frugiperda*.

## Simple Summary

*Calosoma chinense* Kirby, known as the Big Appetite King, is widely distributed in the Huang-Huai-Hai summer corn area. In 2019, *Spodoptera frugiperda* (J. E. Smith) invaded China, primarily feeding on corn. Developing local natural enemy insects has become an urgent task. This report studied the predation capacity of the third-instar larvae and adults of both sexes against the first- to sixth-instar larvae, pupae, and male and female adults of *S. frugiperda*. The predation preference for different developmental stages of *S. frugiperda* was further examined. The results indicated that *C. chinense* exhibited a strong predation ability on *S. frugiperda*. Our study elucidated that *C. chinense* has the potential for application in the biological control of *S. frugiperda.*

## 1. Introduction

*Spodoptera frugiperda* (J. E. Smith), a member of the Lepidoptera order and the Noctuidae family, commonly known as the fall armyworm (FAW), has emerged as a significant invasive pest globally in recent years. The FAW was officially reported to have invaded China after January 2019 [1]. It has progressively spread to southern provinces and is advancing northward, posing a substantial threat to grain production and agricultural development and even endangering national food security.

The FAW is preyed upon by numerous natural enemies, including species from the orders of Coleoptera, Hymenoptera, Dermaptera, and Hemiptera [2,3,4]. Apart from a few studies [5,6,7], little is known about the impact of *C. chinense* on reducing *S. frugiperda* populations in agricultural systems. Since its invasion of China, the use of pesticides for emergency control has increased, and there has been reduced susceptibility to several insecticides that have been used for decades in its native range. Consequently, developing local natural enemy resources for ecological management is a crucial strategy for the sustainable prevention and control of *S. frugiperda*.

*C. chinense*, belonging to the Carabidae family within the Coleoptera order, is widely distributed across various regions of China, including Heilongjiang, Liaoning, Inner Mongolia Autonomous Region, Ningxia Hui Autonomous Region, Gansu, Hebei, Shanxi, Shandong, Jiangsu, Anhui, Zhejiang, Hubei, Jiangxi, Fujian, Sichuan, and Yunnan. Understanding the interactions between *C. chinense* and its native natural enemies is vital to devising effective control measures. In this study, we aim to contribute to the existing knowledge by investigating the potential role of one such natural enemy in mitigating the pest’s impact. By exploring the ecological relationships and the effectiveness of natural predators, we can pave the way for more environmentally friendly and sustainable pest management practices.

We established an experimental predator population of *C. chinense* in the laboratory. The characteristics of *C. chinense* were further confirmed with morphological and molecular methods. The predation behaviors and capabilities of the first- to third-instar larvae, as well as male and female adults of *C. chinensis*, with respect to the larvae, pupae, and adults of *S. frugiperda* were elucidated, and their predation preferences were further evaluated.

## 2. Materials and Methods

### 2.1. Predators and Prey

Larvae of the proposed predator *C. chinense* and fifth-instar larvae of *S. frugiperda* were collected from a corn field located at (34.43 N, 111.66 E) in Wangyao Village, Luoning County, Henan Province, China, in June 2021. Both predators and preys were reared under conditions of 28 ± 0.5 °C, a relative humidity of 70 ± 5%, and a 16L:8D photoperiod. The *S. frugiperda* larvae were reared for over three generations by using an artificial diet [8]. The larvae and adults of *C. chinense* were fed on *S. frugiperda* larvae for two generations. Prior to the predation experiment, first- to third-instar larvae and both male and female adults of *C. chinensis* were subjected to a 24 h starvation period.

### 2.2. Morphological and Molecular Identification of C. chinense

The eggs, larvae, pupae, female adults, and male adults of *C. chinense* were observed using the 3D Microscope Osmic Micro 3DM-HD202WF, Shenzhen Aos Micro Optical Instrument Co., Ltd., Shenzhen, China, The morphological characteristics of the eggs, larvae, pupae, and adults were examined in accordance with the descriptions provided by Yu in 1982 [9].

Genomic DNA was extracted from 300 eggs, with one individual each from the first- to third-instar larvae, pupae, female adults, and male adults of the F1 generation post-adult emergence. The target samples were rinsed with distilled water, ground with liquid nitrogen, and deposited in a 1.5 mL centrifuge tube. The genomic DNA was prepared using the Trans Direct Animal Tissue PCR Kit (Beijing Full Jin Sheng Technology Co., Ltd., Beijing, China), following the manufacturer’s instructions. Briefly, 40 μL of AD1 buffer and 10 μL of AD2 buffer were mixed in the centrifuge tube, and the samples were thoroughly ground by using a grinding rod and left to stand at room temperature for 10 min; then, 40 μL of AD3 buffer was added and samples were stored at −20 °C for PCR use.

From the COI gene region of the mitochondrial DNA, approximately 700 base pairs (bp) were successfully amplified using a combination of two designed primers [10]:

LCO1490: 5′-GGTCAACAAAT CATAAAGATATTGG-3′,
HCO2198: 5′-TAAACTTCAGGGTGACCAAAAAATCA-3′.

The PCR reaction system was 50 μL in volume, consisting of 2 μL of DNA template, 4 μL of dNTPs (2.5 mmol·μL^−1^), 2 μL of upstream and downstream primers (20 μM each), 5 μL of 10 × PCR buffer (containing Mg^2+^), 0.25 μL of Taq DNA polymerase (5 U·μL^−1^), and 34.75 μL of ddH2O. The PCR reaction conditions were as follows: initial denaturation at 94 °C for 1 min; followed by 30 cycles of 94 °C for 30 s, 50 °C for 30 s, and 72 °C for 1 min, with a final extension at 72 °C for 8 min. The amplified product was detected as a single bright band with electrophoresis. The amplified products were then purified and sequenced by Sangon Biotech (Shanghai) Co., Ltd. (Shanghai, China).

Sequence alignment was performed by using Standard Nucleotide BLAST+ 2.16.0 (https://blast.ncbi.nlm.nih.gov/Blast.cgi?PROGRAM=blastn&PAGE_TYPE=BlastSearch&BLAST_SPEC=&LINK_LOC=blasttab&LAST_PAGE=blastn) (accessed on 16 December 2024). Sequence divergences were evaluated by using the Kimura two-parameter (K2P) distance model [11]. The graphic representation of the divergence patterns between the species was provided by calculating the neighbor-joining (NJ) trees based on the K2P distances. Bootstrapping was executed in MEGA12 with 1000 replications [12]. Further validation was conducted by using the NJ method, which also consistently grouped the target samples with *C. chinense*, further reinforcing our identification.

### 2.3. Functional Responses of Predation by Third-Instar Larvae, and Male and Female Adults of C. chinense on First- to Sixth-Instar Larvae and Adults of S. frugiperda

The experiment was conducted in a culture dish with a diameter of 15 cm and a height of 9.0 cm. The predation densities for different instars of *S. frugiperda* larvae were determined based on the following: for first-instar larvae, densities were established at 60, 120, 180, 240, and 300 individuals per dish; for second-instar larvae, 40, 60, 80, 100, and 120 larvae per dish; for third-instar larvae, 5, 10, 20, 40, and 60 larvae per dish; for fourth-instar larvae, 10, 20, 30, 40, and 50 larvae per dish; for fifth-instar larvae, 5, 10, 15, 20, and 25 larvae per dish; and for sixth-instar larvae, 1, 3, 5, 7, and 9 individuals per dish. Each treatment was implemented with a control group and repeated 20 times, and the natural mortality rate was documented. After a period of 24 h, the number of surviving larvae at different densities was observed and recorded. An artificial diet was provided in the Petri dishes for feeding the *S. frugiperda* larvae. Each treatment included the corresponding density of *S. frugiperda* as a blank control, and surviving individuals were assessed after 24 h. All experimental conditions remained consistent throughout.

### 2.4. Predatory Capacity of C. chinense Against First- to Sixth-Instar Larvae, Pupae, and Adult Males and Females of S. frugiperda

Under laboratory conditions, first- to third-instar larvae of *C. chinense*, together with male and female adults that emerged and completed feeding on the same day, were each placed individually in transparent plastic cages measuring 300 mL (120 mm × 90 mm × 43 mm). To mimic ecological feeding conditions, the cages were filled with 3 cm of moist sand. Based on the results from the preliminary experiment, on the second day, *S. frugiperda* individuals in various developmental stages (larvae, pupae, and adults) were introduced into each cage. After initial laboratory trials, 50 individuals of *S. frugiperda*, including first- to sixth-instar larvae, pupae, and both male and female adults, along with one individual of *C. chinense* per stage as indicated above, were added to each cage. Additionally, an artificial diet was provided as food for *S. frugiperda* larvae. Adults were provided with a wick-shaped honeypot containing a 5% honey solution for nourishment. Each treatment included a control group in which *C. chinense* was kept without any prey and was only provided with defatted cotton balls soaked in sterilized distilled water to sustain its life, enabling the assessment of natural mortality rates for both predators and prey. Each treatment was repeated 20 times, and the number of predation and cannibalism instances were counted.

The predation behavior of *S. frugiperda* on *C. chinense* was monitored by using a video microscope (Osmic Micro 3DM-HD202WF, Shenzhen Osmic Micro Optical Instrument Co., Ltd., Shenzhen, China). Twenty-four hours later, the natural deaths and predation instances among *S. frugiperda* and the natural deaths and self-mutilation instances among *C. chinense* under various treatments were documented. The corrected predation rate for each prey was calculated as follows: number of *S. frugiperda* deaths in the treatment group minus number of *S. frugiperda* deaths in the control group.

### 2.5. Prey Selection by Third-Instar Larvae and Adult Males and Females of C. chinense Among Larval, Pupal, and Adult Stages of S. frugiperda

Based on preliminary experiments, the predation capabilities of third-instar larvae and both male and female adults of *C. chinensis* were found to be strong, making them ideal candidates for the release of natural enemies under corn field conditions. Consequently, the predation preferences of these third-instar larvae and adults of *C. chinensis* towards the larvae, pupae, and adults of *S. frugiperda* were assessed. The selected third-instar larvae and female and male adults were subjected to a period of starvation for 24 h in the cage with the same specifications as previously mentioned. Following this preliminary experiment, a mixture of 10 larvae from various instars (including those fed an artificial diet), pupae, and both male and female adults (with a honeypot containing a 5% honey nutrient solution for adults) of *S. frugiperda* were placed in a rectangular insect cage (dimensions: 250 mm × 180 mm × 100 mm) and introduced to the starved *C. chinensis*, with one individual per cage. The larvae were maintained in an artificial climate chamber at a temperature of (27.2 ± 0.5) °C, with a light cycle of 16 h of light followed by 8 h of darkness, and a relative humidity of (80 ± 5)%. From 8:00 to 12:00 in the following day, the quantity of *S. frugiperda* consumed by *C. chinensis* in various life stages in each cage was monitored and recorded, with each treatment being repeated 20 times. The control treatments were consistent with Section 2.3 above. The predation preference of *C. chinensis* for different developmental stages of *S. frugiperda* was determined by using the preference index value Ci, calculated as follows: Ci = (Qi − Fi)/(Qi + Fi) [13], where Ci represents the predator’s preference index for the prey, Qi is the proportion of predators targeting prey “i”, and Fi indicates the proportion of prey “i” in the environment. In this context, N_i_ denotes the number of prey “i” in the environment, and Na_i_ is the number of predators consuming prey “i”, leading to F_i_ = N_i_/∑N_i_ and Q_i_ = Na_i_/∑Na_i_. A positive preference for the first prey is indicated by 0 < C_i_ < 1, while a negative preference is represented by −1 < C_i_ < 0. Duncan’s Multiple Range Test was used to compared between different treatments.

### 2.6. Statistics and Analysis

The functional response model, developed by Rogers in 1972 [14], was subsequently used to describe how consumption in *C. chinense* varied with the availability of *S. frugiperda*. The functional response is described by the equation Na = aNTr/(1 + aThN), whereas the search effect is represented by S = a/(1 + aThN). In these equations, Na denotes the number of each instar larva of *S. frugiperda*, N represents the density of each instar larva, “a” indicates the predator’s instantaneous attack rate on the prey, Tr denotes the total duration of the predation test (here, 24 h), Th is the time taken by *C. chinensis* to consume a single *S. frugiperda* larva, and *S* is the search effect [14]. Initially, the data were processed using Comma-Separated Values (CSVs), followed by ANOVA and pair-wise comparison of the mean consumption under the different treatments to determine significant differences conducted with R-4.4.2 software(R: The R Project for Statistical Computing). Data in the treatments were represented as mean ± SE. Duncan’s new multiple range were used to rank the means.

## 3. Results

### 3.1. Morphological and Molecular Identification

#### 3.1.1. Morphological Identification

During the period from May to October in 2021, we discovered a specific type of carabid larva in Luoning County, Luoyang City, Henan Province of China. After feeding these carabid larvae to adults in the laboratory, we observed that the dorsal side of both male and female mature insects was dark with a coppery sheen interspersed with spots. The insects had rectangular elytra, each adorned with four rows of golden coarse spots. Based on the characteristics mentioned in Liang et al., 2000, these beetle larvae were identified as belonging to the Carabidae family [9].

**Figure 1:**
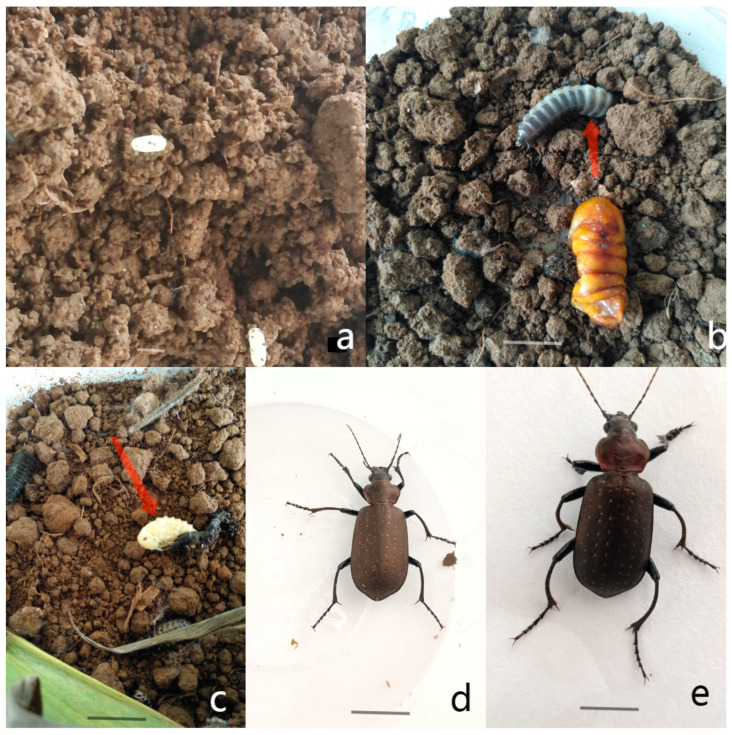
Morphological characteristics of *Calosoma chinense* Kirby. (**a**): Eggs. (**b**): Larva of the second stage. (**c**): Pupa. (**d**): Female adult. (**e**): Male adult. Scale bar = 1mm, with the arrow in the figure indicating the target *Calosoma chinense* state.

*C. chinense* female adults laid an average of 50.2 ± 18.5 eggs per female and typically spawned 2.35 ± 6.2 times during the breeding season. The eggs were deposited individually and were found approximately 7.75 ± 3.24 cm beneath the soil surface. Freshly laid eggs appeared milky white, transitioning to light yellow after one day and eventually dark brown. Prior to hatching, the egg shape was is oval, with an average length of 1.29 ± 0.18 mm and a width of 0.98 ± 0.12 mm. Upon hatching, the eggs were approximately 2.5 mm in length and exhibited a similar coloration to the adults, albeit with a more translucent appearance. The morphological characteristics of both eggs and nymphs were consistent with previous descriptions in the literature [7] (Figure 1a).

The larvae of *C. chinense* were slender, elongated, and black, exhibiting agility and liveliness. They tended to be active at night and remained concealed during the day. Throughout their larval stage, they underwent three instars. The older, mature larvae first constructed a pupal chamber before entering the pupal stage (Figure 1b).

The pupal chamber was typically constructed in a sheltered and secure location, such as under leaves or within the soil. Once inside the chamber, the larvae underwent metamorphosis, transforming into pupae. The pupal stage was characterized by a non-feeding, dormant period, during which the larvae underwent significant physiological changes in preparation for adulthood. Upon completion of metamorphosis, the adult insects emerged from the pupal chamber, ready to begin their reproductive cycle. The distinct morphological characteristics and behavioral patterns of *C. chinense* larvae and pupae provided valuable insights into their life cycle and ecological adaptations (Figure 1c).

*C. chinense* typically exhibited a bronze coloration with a prominent metallic sheen. Small, irregularly arranged particles could be observed between the rows of the elytra, measuring approximately 1 cm in length. In male adults, the first one-to-three tarsal segments of the foreleg and the first tarsal segment of the mid-leg were slightly widened, and there was a hairy pad on the abdomen. In contrast, female adults lacked these characteristics present in males.

Additionally, the antennae of *C. chinense* were relatively long and slender, extending beyond the elytra when at rest. The head was characterized by a prominent, convex forehead and large, compound eyes. The pronotum, which is the area immediately behind the head, was broad and slightly convex, with a distinct median keel running longitudinally. The elytra, which are the hardened forewings of the insect, were smooth and showed a distinct longitudinal striation pattern. Upon closer examination, minute punctures could be observed on the surface of the elytra, contributing to its overall texture.

The differences between male and female adults were as follows: Females were typically slightly larger than males. Male antennae were relatively longer, with a more developed terminal segment. Males have a narrower prothoracic shield, whereas females possess a wider and more rounded one. The female abdomen ended in a relatively blunt and rounded shape, while the male’s is slightly pointed. Regarding foot features, the male forefoot’s tarsus was more developed and was used to grasp the female. Microscopic observation revealed that the aedeagus (with a specific chitinization structure) and endophallus in male adults and the ovipositor, dorsal ovipositor valve, and genital chamber in female adults could significantly facilitate the differentiation between male and female adults (Figure 1d,e).

#### 3.1.2. Molecular Identification

**Figure 2:**
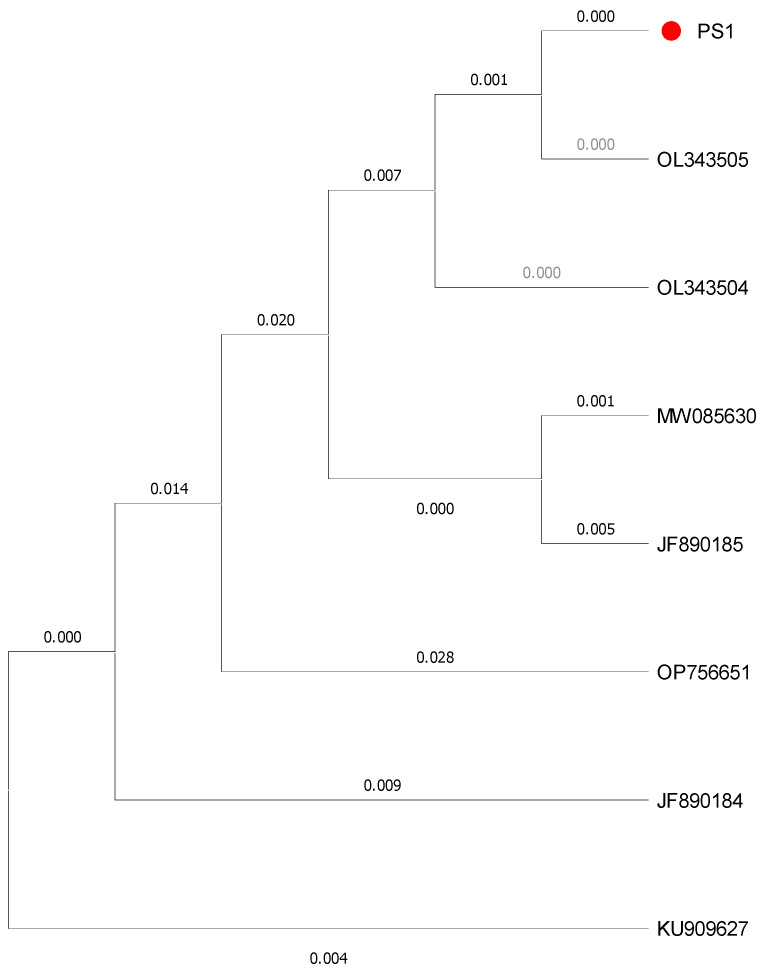
The Maximum Likelihood tree of *Calosoma chinense* Kirby(PS1) based on partial COI haplotype sequences. PS1: The species to be identified. OL343505: *Campalita chinense* Kirby (GenBank: OL343505.1); OL343504: *Campalita chinense* Kirby (GenBank: OL343504.1); MW085630: *Campalita chinense* Kirby (GenBank: MW085630.1); JF890185: *Coleoptera* sp. (GenBank: JF890185.1); KU909627: *Campalita maderae* Fabricius (GenBank: KU909627.1); JF890184: *Coleoptera* sp. (GenBank: JF890184.1); and OP756651: *Calosoma auropunctatum* Herbst. The numbers above and below the nodes denote bootstrap support. The red dot represents the species to be identified in this study.

Target samples were obtained with PCR amplification. The mitochondrial CO I sequence measured 700 bp in length. This sequence was compared with those in the GenBank and BOLD databases, revealing a 100% similarity with the *C. chinense* COI sequence (GenBank: OL343503.1). Based on the analysis results from Clustal W multiple sequence alignment, we assessed branch support in our ML trees by using non-parametric bootstrapping with heuristic searches of 1000 replications. The target samples and *C. chinense* clustered into a single branch, distinctly separated from other related species. It can be concluded that the target samples were *C. chinense* (Figure 2).

The genetic distance analysis revealed minimal divergence between our target samples and the reference *C. chinense* sequence, supporting the molecular evidence for species identification. Overall, the integration of morphological and molecular approaches provided robust evidence for confirming the identity of the target samples as *C. chinense*.

### 3.2. The Predation Behavior and Predatory Functional Response of C. chinense to Larvae of S. frugiperda

#### 3.2.1. The Predation Behavior of *C. chinense* Against *S. frugiperda*

**Figure 3:**
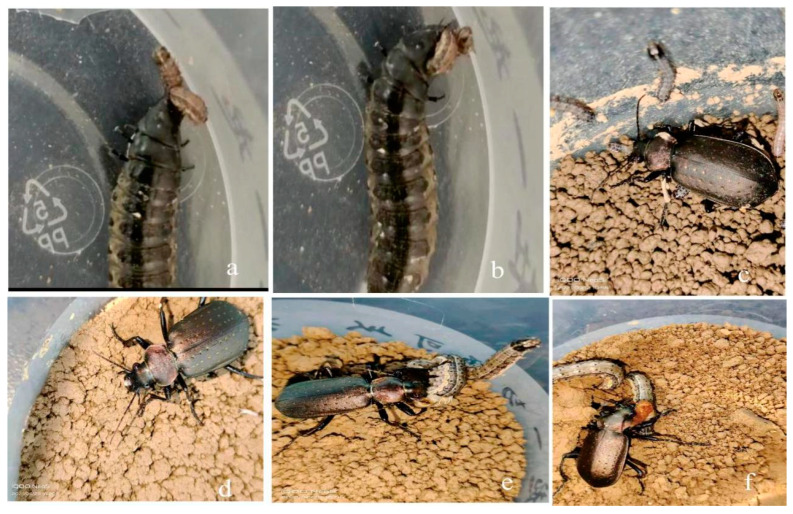
The third-instar larvae and both female and male adults of *Calosoma chinense* Kirby preyed upon the larvae of *Spodoptera frugiperda* (J. E. Smith). (**a**): A third-instar larva of *C. chinense* clamping onto a fifth-instar larva of *S. frugiperda*. (**b**): After clamping, the third-instar larva of *C. chinense* feeding on the fifth-instar larva of *S. frugiperda*. (**c**): A female adult of *C. chinense* preying on third-instar larvae of *S. frugiperda*. (**d**): A male adult of *C. chinense* preying on third-instar larvae of *S. frugiperda*. (**e**): A female adult of *C. chinense* preying on fifth-instar larvae of *S. frugiperda*. (**f**): A male adult of *C. chinense* preying on fifth-instar larvae of *S. frugiperda*.

The laboratory predation experiments showed that the first- to third-instar larvae and both male and female adults of *C. chinense* possessed significant predatory abilities against first- to sixth-instar larvae, pupae, and adults of *S. frugiperda*. The first- to third-instar larvae of *C. chinense* were capable of preying on *S. frugiperda* larvae across various instars and can burrow into the soil to search for prey. The predation process consists of five steps: searching, tempting, attacking, biting and eating. During predation, *C. chinense* used its upper jaws to grasp the *S. frugiperda* larvae. The first- to third-instar *S. frugiperda* larvae were entirely consumed by *C. chinense* before the predator moved on to other targets. For fourth- to fifth-instar prey, *C. chinense* immobilized the larvae by using their appendages and mouthparts until the prey ceases to struggle. Then, *C. chinense* punctured the epidermis to extract body fluids, gradually feeding on the entire body of *S. frugiperda*. *C. chinense* typically consumed the soft parts of the prey’s body, leaving only hard remnants. There were no significant behavioral differences between larvae and adults. Remarkably, if hungry, the first- to third-instar larvae of *C. chinense* could fully consume the entire body of sixth-instar *S. frugiperda* larvae, which were much larger than themselves, before targeting another prey (Figure 3).

A molecular analysis was conducted to further confirm the prey–predator relationship observed. The PCR amplification of specific genetic markers (COI) was performed on samples collected from the gut content of both predators and their respective prey. The sequence analysis revealed high similarity between the prey larvae’s DNA and that of the larvae of *C. chinense*, indicating a potential dietary preference or specificity. Additionally, the PCR results from *S. frugiperda* larvae that had preyed upon *C. chinense* larvae showed traces of *C. chinense* DNA, thus confirming the predation events observed during the experiments.

During the predation experiments, both larvae and adults of *C. chinense* were observed to emit unpleasant white secretions. When satiated, they would typically extracted only a small amount of body fluid from the prey larvae after gnawing the cuticle, then abandoned the prey to search for other sources of food. Notably, first- to second-instar *C. chinense* larvae were occasionally preyed upon by fifth- to sixth-instar *S. frugiperda* larvae.

#### 3.2.2. Predatory Functional Response of Third-Instar Larvae and Female and Male Adults of *C. chinense* to First- to Sixth-Instar Larvae of *S. frugiperda*

A type II functional response was exhibited by the third larvae, as well as female and male adults, of *C. chinense* towards the first to sixth-instar larvae of *S. frugiperda*. This response was modeled by using Holling’s disc equation (Table 1). As prey density increased, so did the consumption by *C. chinense*. However, once the larvae of *S. frugiperda* reached a certain density, the predation rate plateaued.

Among the third larvae and female and male adults of *C. chinense*, the female adults exhibited the highest daily consumption, with a maximum of 1666.67 recorded during the first larval stage of *S. frugiperda*, followed by male adults and third-instar larvae. *C. chinense* consumed more prey as the instar of the *S. frugiperda* larvae decreased. The attack rate and handling time also varied, with the attack rate increasing and handling time first increasing and then decreasing.

#### 3.2.3. Ability of *C. chinense* to predate on Larvae, Pupae, and Adults of *S. frugiperda*

The results indicated that the first- to third-instar larvae, along with male and female adults, of *C. chinense* were capable of preying on larvae, pupae, and adults of both sexes of *S. frugiperda*. The predation rates of *C. chinense* in various developmental stages for *S. frugiperda* larvae of the same age diminished as the instar stage of *S. frugiperda* increased. Among the various developmental stages of the predators, female *C. chinense* adults exhibited the highest predatory capacity, consuming an average of 38.90 ± 0.79 first-instar *S. frugiperda* larvae daily, which was 2.17 times greater than their consumption of sixth-instar larvae (17.90 ± 0.79). Male adults of *C. chinense* followed closely, with a daily predation number of 33.90 ± 1.89 on first-instar larvae. Additionally, the first-instar larvae of *C. chinense* also demonstrated a particular predation ability, with an average daily consumption of 6.95 ± 0.88 first-instar *S. frugiperda* larvae and 0.40 ± 0.50 of *S. frugiperda* pupae. In conclusion, *C. chinense* displayed effective predation on both *S. frugiperda* larvae and adults, particularly preferring the younger larvae of *S. frugiperda* (Table 2).

Furthermore, the predation by *C. chinense* in various instar stages on *S. frugiperda* larvae of the same instar increased with the developmental stage. For *S. frugiperda* larvae from the first instar to the third instar, female *C. chinense* adults exhibited the highest predation efficiency, followed by male adults and then third-instar larvae. However, no significant differences were observed in the predation rates of male and female adults of *C. chinense* and third-instar larvae for the fourth- to sixth-instar larvae or male and female adults of *S. frugiperda* (Table 2).

#### 3.2.4. Predatory Selectivity of Third-Instar Larvae and Male and Female Adults of *C. chinense* for *S. frugiperda*

Among the mixed prey of different-instar larvae, pupae, female adults, and male adults of *S. frugiperda*, individuals in all developmental stages could be preyed upon by the third-instar larvae, female adults, and male adults of *C. chinensis*, which could even consume the hard puparium. However, *C. chinensis* preferred young larvae, with the highest predation being observed in female adults on the first-instar larvae of *S. frugiperda*, averaging 10.0 ± 0.1 individuals per day. The predation quantity among adults was lower than that in larvae, and the third-instar larvae consumed the fewest female adults of *S. frugiperda*, with an average of 2.5 ± 1.4 individuals per day. Throughout all stages, *C. chinensis* showed a positive preference for first- to fourth-instar larvae of *S. frugiperda* and a negative preference for fifth- to sixth-instar larvae, as well as male and female adults, of *S. frugiperda* (Table 3).

## 4. Discussion

The life cycle of *C. chinense* in Henan Province, China, is typically around one year. Upon collecting young larvae in the field, even professionals face challenges in accurately identifying them due to the absence of distinct verified diagnostic features. Generally, laboratory rearing is necessary until the larvae mature into adults, a process that could extend from several months to a year, thus prolonging the identification period.To overcome this obstacle, in our study, we employed DNA barcoding technology, utilizing the mitochondrial CO I gene as a molecular marker to identify the collected carabids. This approach significantly reduced both the time and cost associated with identification. Combined with morphological characteristics, the identification results were not only accurate but also objective and straightforward.

Our study highlights the importance of integrating molecular techniques with traditional morphological methods. While morphological identification relies heavily on the observer’s expertise and experience, which can be subjective and prone to errors, DNA barcoding offers a more standardized and reproducible approach. The use of the mitochondrial CO I gene as a molecular marker has been widely accepted in the field of taxonomy due to its high variability and conservation across species. This made it an ideal choice for our study, allowing for rapid and reliable identification of the Carabidae species collected.

Furthermore, the application of DNA barcoding in our study demonstrates its potential as a powerful tool for biodiversity conservation and ecological research [15]. By enabling rapid and accurate identification of insects, scientists can better understand their distribution, abundance, and interactions within ecosystems. This information is crucial for formulating effective conservation strategies and managing natural resources sustainably. In conclusion, our study underscores the value of combining molecular and morphological methods for insect identification and the potential of DNA barcoding in advancing ecological research.

An invasive insect species, such as *S. frugiperda*, often lacks effective natural predators in its new environment, making it prone to outbreaks that can severely impact agricultural production. Over the past six years, *S. frugiperda* has rapidly spread across most regions of China. The use of local natural enemies to control *S. frugiperda* has become a crucial research topic. *C. chinense* is a voracious predator [16], and the predatory earwig *Doru luteipes* Scudder has been considered for augmentative release in maize fields as a natural enemy. Releasing one pair of *D. luteipes* per corn plant could control the population of *S. frugiperda* and increase maize production by 7% [17,18]. These predatory natural enemies have played a significant role in regulating the population of *S. frugiperda* in the natural environment.

However, it is important to note that the effectiveness of natural predators can be influenced by various factors, such as climate, habitat conditions, and the presence of other pests, such as *Episyrphus balteatus* de Geer, (Diptera: Syrphidae) [19]; understanding how to utilize natural predators effectively is a new approach that we should endeavor to adopt. Therefore, a comprehensive pest management strategy that integrates biological control with other methods, such as cultural practices and chemical treatments, may be necessary to ensure optimal control of *S. frugiperda* and protect agricultural production from losses due to this pest. Furthermore, ongoing research is crucial to identifying additional natural enemies and developing more effective and environmentally friendly control measures against this invasive insect species.

Predicting the impact of local predators on invasive species is important to prioritizing control interventions. Functional response experiments, which examine the consumption of local predators in relation to prey density, represent a useful method for assessing the potential strength of novel predator–prey relationships. However, such experiments are often conducted without consideration of the different developmental stages of predators to reduce invasion risk. Here, we determined the functional responses of third instars and males and females of *C. chinense*, a generalist predator, feeding on the global invader (*S. frugiperda*) to assess whether individuals in the third instar and adults of both sexes have a similar impact potential. We also examined potential correlates of predation behavior by measuring prey choice. Third larvae and adults of both sexes of the predator displayed a type II hyperbolic functional response, which indicates that they can affect *S. frugiperda* populations at low prey densities; they exhibited some differences in foraging behavior: females had slightly lower attack rates, which were not linked to sex differences in movement, and slightly longer handling times, which were not linked to sex differences in prey choice. These small, non-significant differences nevertheless resulted in significantly greater functional response ratios, which were used to predict the ecological impact of the invasive species. There was a significant difference in the proportion of prey consumed between males and females, but the latter had a shorter handling time. Taken together, these results and stage-level modeling suggest that trying to evaluate the potential impact of *C. chinense* on *S. frugiperda* populations by sampling only one of the stages of the predator could lead to wrong estimation results, even in populations that have male-biased sex ratios. The consumer stage of the predator might generally be an important characteristic to consider when using functional response experiments to estimate the control effect on new invasive species, especially those with marked migratory characteristics that affect foraging.

In our study, the predator *C. chinense* exhibited a type II functional response when feeding on *S. frugiperda* in all life stages. The number of *S. frugiperda* individuals consumed increased with the increase in *C. chinense* density until prey consumption reached saturation. In another report on predatory actions against *S. frugiperda* [20] also observed this type of functional response in Dermapteran *Labidura riparia* Pallas to the same prey. This functional response type is ideal for biological control because predators can detect and attack their prey at low densitie

However, it is important to note that the effectiveness of biological control using *C. chinense* or other predators may depend on various environmental factors, such as climate, habitat structure, and the availability of alternative prey species. For instance, changes in temperature and precipitation patterns can influence the growth and reproduction rates of both predators and prey, potentially altering their interaction dynamics. Furthermore, the presence of competing predators or parasites may also affect the ability of *C. chinense* to effectively control *S. frugiperda* populations Therefore, future studies should consider these environmental variables when assessing the potential of *C. chinense* for the biological control of *S. frugiperda* [21].

Our study demonstrated that *C. chinense* exhibited effective predation on *S. frugiperda*, with both its larvae and adults being able to consume *S. frugiperda* in different insect stages. It further indicated that employing *C. chinense* can lead to the immediate death of young *S. frugiperda* larvae, pupae, and adults, outperforming parasitic natural enemies, whose victims often remain able to move and continue feeding for a period after post-parasitism. *C. chinense* had also been documented to prey on various pests, including larvae of *Mythimna separata* Walker, *Spodoptera litura* Fabricius, *Agrotis ypsilon* Rottemberg, *A. segetum*, *A. tokionis*, *Dolerus tritici* Chu, and *Pieris rapae* Linnaeus [22]. Therefore, *C. chinense* not only serves as a natural enemy of the invasive *S. frugiperda* but also plays a role in controlling other common pest species. Although *C. chinense* in all growth stages could control *S. frugiperda* in the laboratory, further study is needed to determine the damage it may cause to maize.

This study revealed that as the instar stage of *S. frugiperda* larvae increased, the population of third-instar larvae of *C. chinensis* diminished steadily, alongside an increase in the duration of prey handling. In instances of approaching satiation, when the predator breached the prey’s epidermis, it tended to extract only a minimal quantity of body fluid from the prey larvae, subsequently abandoning it to seek out other larvae for continued predation. While hunting *S. frugiperda* larvae, both adult and larval *C. chinensis* exhibited defensive behaviors by spraying an unpleasant white secretion when confronted with strong prey that exceeded their own body size, before proceeding to prey upon other prey. This behavior indicated that they, similar to many Coleoptera insects, are not deterred by larger individuals. Chemical defense emerged as the primary means of protection for themselves. The chemical substances produced are generally stored in specialized defense glands, and when provoked, these carabids choose to attack by releasing the substances rather than fleeing. The same phenomenon, which is a normal defensive response, has also been observed in predation on armyworms and *Helicoverpa armigera* (Hübner, 1808) by *Labidura riparia* Pallas [13].

Additionally, our findings highlight the potential impact of prey size on the predation behavior of *C. chinensis*. When confronted with larger *S. frugiperda* larvae, *C. chinensis* exhibited heightened caution, often engaging in more evasive maneuvers before initiating an attack. This suggests that prey size plays a crucial role in determining the predation strategy employed by *C. chinensis*. Moreover, the observed reduction in predator population targeting the third-instar larvae of *S. frugiperda* as they matured could be attributed to the increasing difficulty in overcoming the prey’s defenses, leading predators to shift their focus towards younger, more vulnerable larvae.

It is also noteworthy that the duration of prey handling increased as the instar stage of *S. frugiperda* larvae increased. This could be due to the thicker exoskeleton and stronger defenses developed by the larvae as they matured, making them more challenging to prey on. Consequently, predators had to invest more time and energy into subduing and consuming these larger larvae.

Overall, our study provides valuable insights into the complex interactions between predators and prey, particularly in the context of biological control. Understanding these interactions is crucial to developing effective pest management strategies that leverage the natural predation behaviors of insects, such as *C. chinensis*.

## 5. Conclusions

In this study, the predation capacity of third-instar larvae and adults both of sexes of *C. chinense* with respect to first- to six-instar larvae, pupae, and male and female adults of *S. frugiperda* was elucidated. The predation preference for *S. frugiperda* in different developmental stages was further clarified. Our results show that *C. chinense* in all stages could prey on *S. frugiperda* in various stages, even adults, and especially preferred young larvae, which possess strong predatory capacities.

Among them, female adults of *C. chinense* exhibited the strongest predatory capacity against first-instar *S. frugiperda* larvae. The first-instar larvae of *C. chinense* also demonstrated *S. frugiperda* control ability. The predation ability of *C. chinense* larvae increased with each instar, particularly against first- to third-instar *S. frugiperda* larvae. Female adults of *C. chinense* possessed the strongest predation ability, followed by male adults and then third-instar larvae. However, *C. chinense* displayed a positive preference for *S. frugiperda* larvae from the first instar to the fourth instar while showing a negative preference for fifth- to sixth-instar larvae.

The FAW, a notorious agricultural pest native to South and North America, has emerged as a significant invasive insect globally in recent decades, primarily preying on corn. *C. sinensis* serves as a crucial natural enemy of agricultural pests in the maize fields of the Huang-Huai-Hai region. It preys on numerous lepidopteran larvae and is an important natural enemy of pests, such as armyworms and fall armyworms. The predatory capacity of *C. chinense* is considered ideal, as its daily predation can exceed 28 sixth-instar larvae of *S. frugiperda*. This indicates that *C. chinense* has great potential to be utilized as a biocontrol agent against these voracious pests. On the other hand, *C. chinense* could also pose a threat to *Bombyx mori* Linnaeus if they infiltrate the silk industry [23]. The FAW, originating from the Americas, has now “settled” in China. Strategies for the management of this species will need to be developed taking into account food resources from non-crop habitats and the utilization of shelters to sustain natural enemies, such as *C. chinense*, based on open-field experiments.

In the agricultural ecosystem, protecting and utilizing natural enemies of *S. frugiperda* is one of the key strategies for comprehensive pest prevention and control. Integrated pest management, rather than reliance on a single tactic, is the best way to suppress the *S. frugiperda* population.

## Figures and Tables

**Table 1:** Predation functional responses of *Calosoma chinense* Kirby third larvae and female and male adults to larvae and adults of *Spodoptera frugiperda* (J. E. Smith).

Predation Functional Responses		Equation of Predation Functional Response	Correlation Coefficient (R^2^)	Attacking Efficiency (a)	Handling Time (Th) (d)	Predatory Efficacy (a/Th)	Maximum Daily Consumption
*S. frugiperda*	*C. chinense*						
1	3	Na = 1.11 N/(1 + 0.023 N)	0.9851	1.11	0.0015	484.17	434.78
	Female adult	Na = 1.124 N/(1 + 0.0007 N)	0.8757	1.12	0.0006	1873.92	1666.67
	Male adult	Na = 0.93 N/(1 + 0.0256 N)	0.9374	0.93	0.0021	612.23	476.19
2	3	Na = 0.733 N/(1 + 0.171 N)	0.9885	0.73	0.0105	69.82	95.24
	Female adult	Na = 1.23 N/(1 + 0.004 N)	0.9087	1.23	0.0034	715.31	294.12
	Male adult	Na = 1.182 N/(1 + 0.003 N)	0.9132	1.18	0.0024	492.69	416.67
3	3	Na = 0.51 N/(1 + 0.014 N)	0.9268	0.51	0.0279	18.29	35.84
	Female adult	Na = 0.93 N/(1 + 0.0256 N)	0.9164	0.93	0.0218	53.96	45.87
	Male adult	Na = 1.03 N/(1 + 0.026 N)	0.9963	1.03	0.0251	40.95	39.84
4	3	Na = 1.02 N/(1 + 0.1 N)	0.8713	1.02	0.0989	10.27	10.11
	Female adult	Na = 0.93 N/(1 + 0.014 N)	0.9323	0.93	0.0173	55.13	57.80
	Male adult	Na = 1.03 N/(1 + 0.028 N)	0.9704	1.03	0.0276	37.34	36.23
5	3	Na = 1.09 N/(1 + 0.19 N)	0.7603	1.09	0.1737	6.30	5.76
	Female adult	Na = 0.93 N/(1 + 0.014 N)	0.9191	0.93	0.0174	55.08	57.47
	Male adult	Na = 1.03 N/(1 + 0.0286 N)	0.9678	1.03	0.0277	37.30	36.10
6	3	Na = 0.979 N/(1 + 0.065 N)	0.8706	0.98	0.0661	14.81	15.13
	Female adult	Na = 0.93 N/(1 + 0.014 N)	0.946	0.93	0.0146	63.53	68.49
	Male adult	Na = 0.956 N/(1 + 0.048 N)	0.9964	0.96	0.0379	33.23	26.39
Female adults	3	Na = 1.26 N/(1 + 0.294 N)	0.8751	1.26	0.2337	5.39	4.29
	Female adult	Na = 0.733 N/(1 + 0.171 N)	0.9885	0.73	0.0105	69.82	95.24
	Male adult	Na = 1.803 N/(1 + 0.007 N)	0.9424	1.80	0.0127	43.68	78.74
Male adults	3	Na = 0.49 N/(1 + 0.114 N)	0.9277	0.49	0.2337	2.09	4.28
	Female adult	Na = 0.459 N/(1 + 0.019 N)	0.9642	0.46	0.0416	11.04	24.04
	Male adult	Na = 0.296 N/(1 + 0.023 N)	0.936	0.30	0.0792	3.74	12.63

Note: Na was the net predation rate by the predator during the hunting time period, a was the instantaneous attack rate, N was the density of prey, Th was the time required to prey on a larva or egg (handling time). a/Th (the theoretical maximum number of prey consumed per day; number); a (per day); Th (days); d (day).

**Table 2:** Daily predatory capacity of *Calosoma chinense* Kirby on larvae, pupae, and adults of *Spodoptera frugiperda* (J. E. Smith).

Larvae and Adults of *C. chinense*	Number of *S. frugiperda* Preyed on by *C. chinense*								
	First-Instar Larvae	Second-Instar Larvae	Third-Instar Larvae	Fourth-Instar Larvae	Fifth-Instar Larvae	Sixth-Instar Larvae	Pupae	Female Adults	Male Adults
First-instar larvae	(6.95 ± 0.88) Ae	(5.95 ± 0.88) Be	(4.95 ± 0.88) Ce	(4.10 ± 0.90) De	(3.05 ± 0.81) Ee	(1.15 ± 0.92) Fe	(0.40 ± 0.50) Gc	(0.80 ± 0.52) FGe	(0.60 ± 0.50) FGd
Second-instar larvae	(10.95 ± 0.89) Ad	(9.95 ± 0.89) Bd	(7.95 ± 0.89) Cd	(6.95 ± 0.89) Dd	(6.10 ± 1.07) Ed	(5.05 ± 0.76) Fd	(1.05 ± 0.89) Gc	(2.00 ± 0.97) Hd	(3.95 ± 0.89) Ic
Third-instar larvae	(25.90 ± 0.79) Ac	(22.75 ± 0.79) Bc	(20.95 ± 0.83) Cc	(19.85 ± 0.81) Dc	(18.80 ± 0.77) Ec	(17.85 ± 0.81) Fc	(14.90 ± 0.79) Hb	(15.90 ± 0.79) Gc	(16.15 ± 0.59) Gb
Female adults	(38.90 ± 0.79) Aa	(37.30 ± 2.32) Ba	(35.85 ± 0.81) Ca	(33.90 ± 0.79) Da	(30.90 ± 0.79) Ea	(27.85 ± 0.81) Fa	(17.90 ± 0.79) Ha	(20.85 ± 0.81) Ga	(20.20 ± 0.40) Ga
Male adults	(33.90 ± 1.89) Ab	(29.20 ± 1.99) Bb	(27.55 ± 1.32) Cb	(25.35 ± 1.53) Db	(23.75 ± 0.79) Eb	(21.80 ± 0.77) Fb	(17.85 ± 1.98) Ha	(20.10 ± 1.74) Gb	(20.20 ± 1.40) Ga

The data in the table are presented as mean ± SE. Different uppercase letters in the same row and different lowercase letters in the same column indicated significant differences in the predatory numbers of *C. chinense* in the same developmental stage preying on *S. frugiperda* in different developmental stages and significant differences in the predatory numbers of *C. chinense* in different developmental stages feeding on *S. frugiperda* in the same developmental stages, respectively (*p* < 0.05, Duncan’s Multiple Range Test).

**Table 3:** Predation preference of third-instar larvae and female and male adults of *Calosoma chinense* Kirby for larvae, pupae, and adults of *Spodoptera frugiperda* (J. E. Smith).

*C. chinense*	*S. frugiperda*																	
	First-Instar LARVAE		Second-Instar Larvae		Third-Instar Larvae		Fourth-Instar Larvae		Fifth-Instar Larvae		Sixth-Instar Larvae		Pupae		Female Adults		Male Adults	
	PN	C_i_	PN	C_i_	PN	C_i_	PN	C_i_	PN	C_i_	PN	C_i_	PN	C_i_	PN	C_i_	PN	C_i_
Third-instar larvae	(9.8 ± 0.3) Aa	0.28 Aa	(9.35 ± 0.9) ABa	0.31 Aa	(8.8 ± 0.5) Ba	0.29 Aa	(6.3 ± 1.3) Cb	0.06 Ba	(5.4 ± 1.3) Db	−0.02 BCb	(4.4 ± 1.3) EFb	−0.13 aCb	(3.4 ± 1.3) Fb	−0.26 Db	(2.5 ± 1.4) FGa	−0.34 DEa	(3.0 ± 1.3) Ga	−0.42Ea
Female adults	(10.0 ± 0.1) Aa	0.19 Ab	(9.8± 0.6) Aa	0.19 Ab	(8.6± 0.1) Ba	0.14 ABb	(8.2 ± 0.8) Ca	0.09 BCa	(7.2 ± 0.8) Da	0.09 Da	(6.3 ±0.7) Ea	−0.04 DEa	(5.7 ±0.9) Ea	−0.1 Ea	(2.5 ± 0.8) Fa	−0.50 Fa	(2.6 ± 08) Fa	−0.50Fa
Male adults	(9.9 ± 0.4) Aa	0.20 Ab	(9.6 ± 0.7) Aa	0.20 Ab	(8.6 ± 1.1) Ba	0.12 ABb	(8.1 ± 0.9) Ba	0.10 Ba	(7.9 ± 0.9) Ba	0.08 Ba	(6.2 ± 0.9) Ca	−0.04 Ca	(5.2 ± 0.7) Da	−0.12 Da	(2.2 ± 0.9) Ea	−0.5 Ea	(2.2 ± 0.5) Ea	−0.5Ea

The data in the table are presented as mean ± SE. Different capital letters in the same row indicate significant differences in the predatory number (preference index) of *C. chinense* in the same developmental stage preying on *S. frugiperda* in different developmental stages (*p* < 0.05), while different lowercase letters in the same column denote significant differences in the predatory number (preference index) among *C. chinense* in different developmental stages feeding on *S. frugiperda* in the same developmental stages (*p* < 0.05) (Duncan’s Multiple Range Test). PN: daily predatory number; Ci: preference index.

## Data Availability

The original contributions presented in the study are included in the article, further inquiries can be directed to the corresponding authors.
